# Low cost and effective reduction of formaldehyde in gross anatomy: long throw nozzles and formaldehyde destruction using InfuTrace™

**DOI:** 10.1007/s11356-020-09961-0

**Published:** 2020-08-11

**Authors:** Sonja Pfeil, Hans Hieke, Petra Brohmann, Monika Wimmer

**Affiliations:** 1 Institute of Anatomy and Cell Biology, Justus-Liebig-University, Aulweg 123, 35392 Giessen, Germany; 2 Department of Real Estate, Construction and Technology, Justus-Liebig-University, Ludwigstraße 23, 35390 Gießen, Germany; 3 Department 35.3, Kassel Regional Council, Ludwig-Mond-Str. 33, 34121 Kassel, Germany; 4 Institute of Anatomy, Johannes Kepler University Linz, Huemerstraße 3-5, 4020 Linz, Austria

**Keywords:** Anatomy dissection course, Formaldehyde fixation, Formaldehyde permissible exposure limits, InfuTrace™, Long throw nozzles, Formaldehyde reduction, Ventilation in anatomy labs

## Abstract

Formaldehyde is extraordinarily effective for fixation of human corpses and is routinely used in embalming solutions in anatomical dissection courses all over the world. High concentrations in vapors emitted from corpses embalmed with formaldehyde make it necessary to reduce the emission from cadavers for fulfilling tightening permissible exposure limits (PEL) worldwide. The study provides possible solutions to a problem faced by many anatomy labs. The emission of 50 human corpses was examined using 240 active personal and stationary samples with sampling tubes placed in the breathing area of probands or directly above the corpses. For measuring formaldehyde exposures along the dissection course, air samples were collected during the progress of dissection. Best results were achieved by a combination of post-embalming treatment with InfuTrace™, a formaldehyde binding solution applied to corpses fixed with 3% formaldehyde, and a modified ventilation system consisting of three long throw nozzles mounted vertically at the ceiling above the longitudinal axis of each dissection table. In this scenario, the inhalative exposure for students and teachers did not exceed 0.1 ppm during muscle dissection and 0.041 ppm during organ dissection, which are both dissection steps linked to high emission rates. The data emphasizes the necessity to use a combination of different methods — chemical polymerization of formaldehyde combined with a modified ventilation system — to reduce formaldehyde air loads far below the German PEL (0.3 ppm) and even the Japanese PEL (0.1 ppm) when using a standard 3%-formaldehyde fixation.

**Electronic supplementary material:**

The online version of this article (10.1007/s11356-020-09961-0) contains supplementary material, which is available to authorized users.

## Introduction

The profound knowledge of human anatomy is an absolute prerequisite for any medical doctor. The dissection of a human corpse is indispensable, as it gives the medical students a realistic impression of the three-dimensional body structures and their varieties (Balta et al. 2017; Brenner 2014; Soares et al. 2018), not forgetting the enormous psychological benefits for the students of learning the management of emotional attitudes and reactions (Arráez-Aybar et al. 2008). Therefore, anatomical departments teach anatomy in gross anatomy classes using preserved corpses of body donors. The preservation of corpses makes them lasting and prevents any risk of infection. One established efficient method for fixation is the use of solutions containing formaldehyde. Formaldehyde is known as an approved chemical for disinfection. The use of formaldehyde ensures optimal results in conservation and disinfection (Brenner 2014). Formaldehyde-based embalmment results in lowest to no microbiological activity over a period of 8 months, while shape and size of organs and vessels are retained (Balta et al. 2018a, b).

In a survey, British and Irish anatomy teachers confirmed the use of formalin for fixation (Balta et al. 2017). Nearly 50% believe that alternative fixations are expensive and do not wish to change their formalin-based embalming techniques (Balta et al. 2017). Yet, new insights in the toxicity of formaldehyde associate the substance with cancer (Rizzi et al. 2016), liver toxicity (Bai et al. 2017), and, in a study concerning occupational formaldehyde exposure, amyotrophic lateral sclerosis (Seals et al. 2017).

In 2004, the International Agency for Research on Cancer (IARC) classified formaldehyde as carcinogenic to humans (IARC 2004). Ten years later, the European Commission classified formaldehyde as carcinogenic (1B) and probably mutagenic (category 2) to humans in the Regulation (EC) No. 605/2014 (EU-Commission 2014). Subsequently, German legislation introduced an official valid permissible exposure limit (PEL) of 0.3 ppm and a short time value of 0.6 ppm to be reached once per working shift (Ausschuss für Gefahrstoffe 2015). In a worldwide comparison, only Japan (0.1 pm/0.2 ppm), the Netherlands (0.12 ppm), and Israel (0.2 ppm) do have stricter regulations (IFA Institut für Arbeitsschutz der Deutschen Gesetzlichen Unfallversicherung 2017) for formaldehyde loads in working places. Previous recordings of formaldehyde air loads in anatomical courses actually revealed concentrations of up to 3.1 ppm (Gurbuz et al. 2016), 3.4 ppm (Risk Assessment Commitee 2012), or even 9.16 ppm (Wright 2012). These values exceed the allowed PELs by far. It is expected that the European Union (EU) will harmonize the occupational exposure limits for formaldehyde use for its member states (ANSES (French Agency for Food, Environmental and Occupational Health Safety), RIVM (Dutch National Institute for Public Health and the Environment) 2019; ECHA - European Chemicals Agency 2019, EU-Commission 2018). Therefore, new and innovative methods for reducing formaldehyde exposure will have to be developed.

New formaldehyde substitutes are available (Al-Hayani et al. 2011; Goyri-o-Neill 2013; Hammer et al. 2012; Turan et al. 2017), but none of them seem to be able to meet the requirements for optimal embalming. Formaldehyde substitutes or embalming solutions containing other harmful substances like phenol with its unacceptable strong odor or glutaraldehyde (German PEL below formaldehyde) are no options. Aminolipine might be a potential alternative fixative for human corpses, giving good results as described by Hirt and published by Neckel et al. (2017). As yet, however, this chemical still awaits approval by the European Chemicals Agency (ECHA).

A different approach to reducing occupational exposure to formaldehyde is modifying airflow around the dissecting table (Demer 2012; Matsuda et al. 2009). Yet, up to now, no really satisfying devices have been developed.

Therefore, a basic research study was initiated with the goal of developing a method for reducing formaldehyde exposure in the dissection lab of the Anatomical Department of the Justus-Liebig-University Giessen. In a first step, the emission process of formaldehyde from formaldehyde-fixed corpses was analyzed. In a second step, a new ventilation system was developed and tested for its effectiveness regarding formaldehyde concentration levels in the dissection lab. Additionally, the effects of reducing the concentration of formaldehyde in the embalming solution were analyzed. Finally, a completely different approach was used by applying a post-embalming treatment of regularly fixed corpses (3% formaldehyde) using a solution which polymerizes free formaldehyde.

## Materials and methods

The experimental study was performed in the dissection lab (Anatomical Department, University Giessen) during regular classes to guarantee real-life conditions.

### Air exchange and climate in the dissection hall

The dimensions of both dissection labs of the Justus-Liebig-University are 17.3 m × 16.8 m (290 m^2^) with a height of 5.5 m and a volume of 1600 m^3^. Both are equipped with twelve dissection tables. The air supply is 13,000 m^3^/h, and the air exhaust accounts for 15,000 m^3^/h. This adds up to 9.4 air changes per hour. Fresh air is delivered by ceiling slot diffusors, and the exhaust air is extracted by ventilation grilles close to the ground. Climate conditions are regulated to 17 °C and approximately 35% relative air humidity. On extremely hot summer days, room temperature rises to 20 °C and relative air humidity reaches 80%.

### Body donors

The anonymized body donors (*n* = 50) included in this study all gave informed consent during lifetime to be part of research projects and student training. The Ethics Committee declared the use of these corpses for scientific studies as legal. Therefore, no separate vote of the local Ethics Committee was required.

In order to obtain valid data for the exposure to formaldehyde in gross anatomy classes, the measurements took place in real-life scenarios. The bulk of data was generated within regular gross anatomy courses in the dissection rooms of the Anatomical Department of the Justus-Liebig-University Giessen. The use of a real-life setting for measuring the realistic exposure load thus imposes a limit on the number of cadavers which can be used for such an analysis: both dissection rooms were equipped with a maximum of 12 cadavers. Apart from this restriction, the high cost for each corpse is also an important limiting factor, as well as the restricted number of donated bodies in stock. Each measurement was repeated three times.

### Embalming methods

All corpses were fixed by perfusion with an embalming solution within 24 h after death.

The perfusion method (closed circuit) is based on gravity. The perfusion stops when the counterpressure in the body equals the force of gravity. The standard volume of the fixation solution is supposed to be about 20 l per cadaver.

The following embalming solutions were tested:

- Embalming solution 1: 3% formaldehyde, 5% phenoxyethanol, 5% glycerin, 62.9% ethanol, deionized water.
- If indicated otherwise, modified solutions were tested for their suitability in preservation or for testing the effects of progressively lowering the formaldehyde concentration for embalmment:
- Embalming solution 2: 2.4% formaldehyde.
- Embalming solution 3:1.85% formaldehyde.
- Embalming solution 4: saturated salt solution (NaCl) with 1.48% formaldehyde.
- Embalming solution 5: 2.14% formaldehyde.
- Embalming solution 6: 1.83% formaldehyde (ingredients see Supplement 1).

Before dissection, the corpses were stored either in tanks filled with a 2% TERRALIN PROTECT solution (Schülke and Mayr 2017) or in bags with a small amount of 2% TERRALIN PROTECT solution.

During the dissection course, the corpses remained in the dissection hall, covered by a plastic foil and a sheet wetted with a formaldehyde-free solution (phenoxyethanol (2200 ml), glycerin (300 ml), thymol (200 ml), Terralin 200 ml, and 2000 ml tap water).

### Long throw nozzle system

**Fig. 1:**
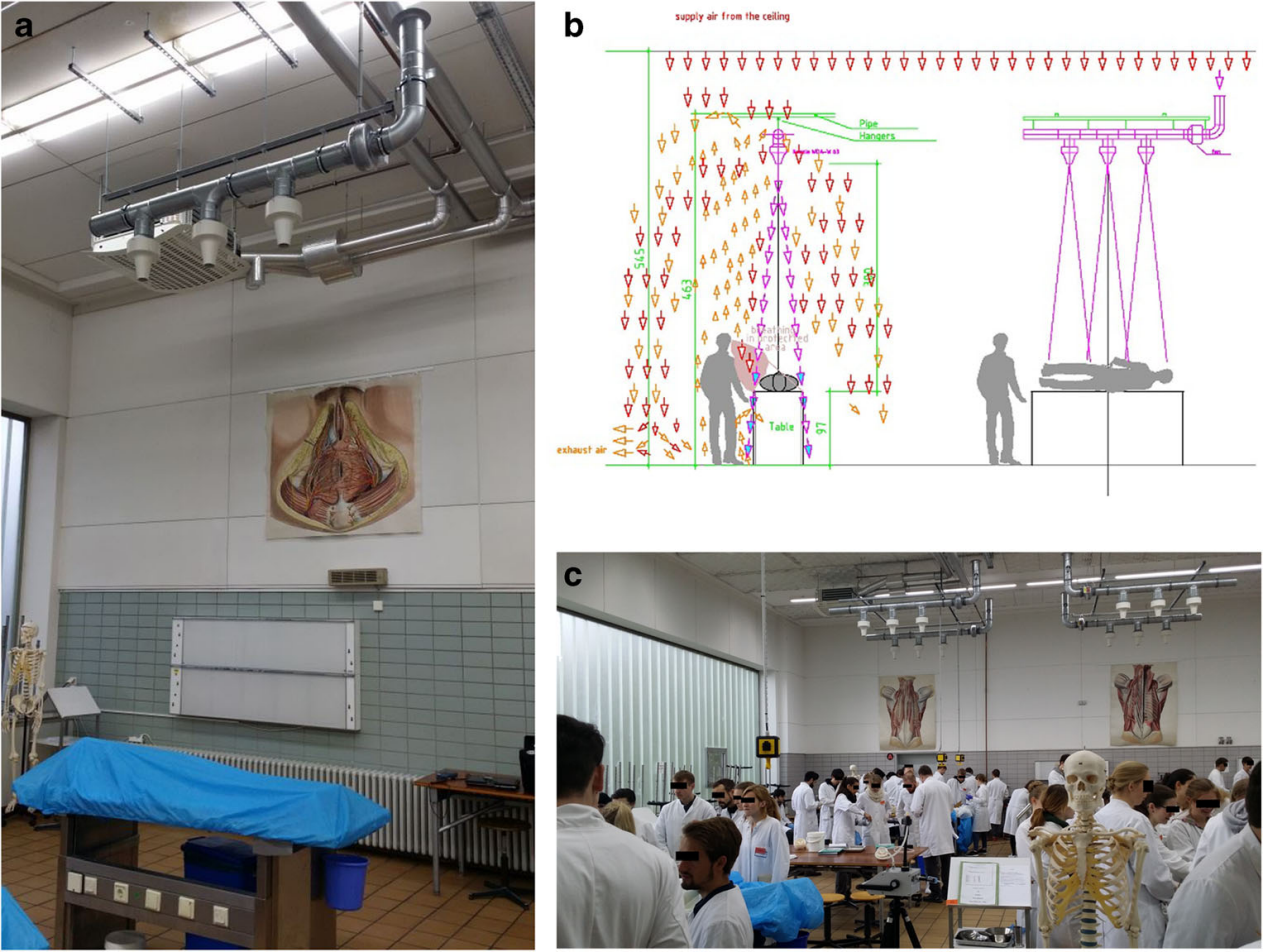
**a**–**c** The long throw nozzle system placed at the ceiling above the longitudinal axis of a dissection table

The ventilation concept required continuous airflow directed at the complete dissection table. For this purpose, a system of three long throw nozzles in a row was constructed and mounted on a steel rail 3 m above each section table along its longitudinal axis (Fig. 1). The long throw nozzle capacity was calculated so as to make sure that the directed airflow hits the corpse and the table surface exclusively. In consequence, the ascending formaldehyde-polluted air is pressed downward to the floor, where it is eliminated by the exhaust system. The air leaves the nozzle conducting system with a velocity of 5.8 m/s and hits the table/corpse with a velocity of 0.6 m/s in the core area of the air jet. Thus, the polluted air, formerly ascending due to thermal updraft, now is barred from ascending. The airflow downward is regulated by an inbuilt damper flap. The tube ventilator with an EC power unit can be regulated continuously from 0 volt to 10 volts. Each nozzle works with a primary pressure of 25 Pa at the nozzle head and a capacity of about 65 m^3^/h. Mixed with indoor air by induction, this adds up to a downward flow volume of 1400 m^3^/h per table. To avoid temperature differences between the air of the hall and the down-streaming air of the nozzles, the required air is aspirated via a tube directed at the ceiling, where ambient and isothermal air is drawn in. On its way down to the table, the jet stream collects ambient air. This further impacts the diluting effect on the emitted formaldehyde.

For measurement of the airflow, a heat wire anemometer Type TA 5 (Airflow Lufttechnik GmbH, Rheinbach, Germany) was used.

**Fig. 2:**
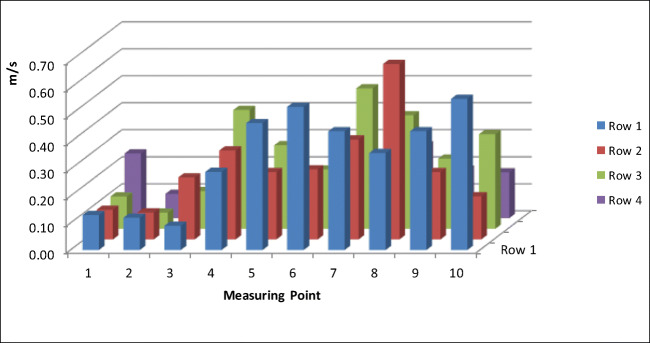
Airflow with running long throw nozzles measured approximately 20 cm above the cadaver placed on the dissection table with highest airflow above the thorax and the low airflow above the head and legs. The additional air jet does not affect comfortable working at the table

To ensure optimal downward flow in the middle of the corpse, where formaldehyde emission will be highest, the middle nozzle was positioned exactly in the middle of the dissection table, and both outer nozzles were fixed at a distance of 0.5 m from the middle nozzle along the longitudinal axis of the table. This results in the highest air speed in the middle (0.6 m/s) and a lower speed (0.15 m/s) at the head and the legs (Fig. 2). The airflow was regulated to guarantee comfortable working without being exposed to draft. It is noteworthy that the airflow velocity downward can be adapted to any distance between the nozzle and the dissection table.

### Materials used for the construction of the long throw nozzles

The materials used for the construction of the long throw nozzles are the following: in-line fan to be installed in circular ducting RR125C (Helios Ventilatoren GmbH + Co KG, Villingen-Schwenningen, Germany), short concentric reducer DN 125-100 (Lindab GmbH, Bargteheide, Germany), regulating damper DN 100 (Lindab, Germany), exhaust air stud DN 100 (Lindab, Germany), volume flow rate measuring unit VMR 100 (Trox Group, Neukirchen-Vluyn, Germany), short concentric reducer DN 200-125 (Lindab, Germany), and long throw nozzle WDA-W 63 (Schako KG, Kolbingen, Germany).

### Formaldehyde sampling and analytics

There are two different methods used for collecting samples: For active sampling in accordance with the NIOSH method 2016 (Tucker 2003), various types of air sampling equipment were used. For active personal sampling the lightweight pumps GSA 2500ex, GSA 250 (GSA Gerätebau GmbH, Ratingen, Germany), GilAir Plus (Sensidyne, St. Petersburg, USA), Buck (A. P. BUCK INC, Orlando, USA), and flow control with TSI Model 4199, 4100 Series (TSI GmbH, Aachen, Germany) were used. DNPH cartridges (Supelco DNPH S10, Sigma-Aldrich, now Merck, catalogue-no.: 21026-U, Germany) were used as adsorbent. Volume flow depended on sampling time and the expected formaldehyde load and varied from 0.33 to 1.5 l per minute. For sampling of ambient air, the personal samplers and the stationary pump BiVOC2 (Holbach company, Wadern, Germany) were used.

A second (passive) sampling method using the passive sampler Radiello® (Florisil® coated with DNPH, Sigma-Aldrich, now Merck, Catalogue-no.: RAD165) was applied. The collected samples were submitted to identical analytics. High amounts of ethanol do not affect sampling and analytics in any way, as stated by Shiraishi (2006).

The air samples were analyzed at the ARGUK-Umweltlabor GmbH (Oberursel, Germany) by HPLC/UV detection after elution with 10 ml acetonitrile. The limit of quantification varied from 0.001 to 0.005 mg/m^3^, dependent on the sample volume.

Air samples from the breathing zone of the probands at the dissection tables were collected in triples: personal samplers were attached to two working persons (students or teacher) in the breathing area. In addition, one stationary sampler was placed above the thorax area of a corpse in breathing height of the probands. The passive sampling devices were placed below the shoulder near the heart of the teachers. For each measuring scenario, the dissection table was surrounded by 8 persons dissecting one corpse. For reasons of comparability, sampling times were 15 min each. Depending on the measuring scenario, a few sampling times were prolonged, e.g., for samples like the brain and spinal cord, used in the neuroanatomy course, as well as for long-term stored corpses used for examinations. Here, stationary measurement setups were used imitating real situations. The ambient air in the dissection lab was collected at two places in the middle of the room between four dissection tables on each experimental day for a time period of 180 min (length of a dissection course). Each measurement was repeated three times.

For statistical analysis, the standard deviation (SD) was calculated, and the data was evaluated using GraphPad Prism. Data is listed in the appendix.

Finally, to confirm the gathered results, air samples were taken by the governmental measuring authority (Regierungspräsidium Kassel, Germany) using the NIOSH method 2016 for air sampling with a volume flow of 0.1 l/min and analytics (HPLC-UV) at the laboratory of the Regierungspräsidium. Personal samplers Gilian LFS-113 (Sensidyne, St. Petersburg, USA) and S 205 (Ametek/Dupont) were used with DNPH Type 226-119 (SKC ANALYT-MTC Messtechnik GmbH, Müllheim, Germany) and flow control via Definer 220-L (Mesa Labs, Lakewood, Colorado, USA). The limit of quantification varied from 0.011 to 0.056 ppm, dependent on the sample volume.

### InfuTrace™ application

InfuTrace™ (American Bio-Safety, Inc., Rocklin, CA, USA), a formaldehyde binding agent, was applied in concentrations of 20% for injection purposes, according to the instructions for use, and in a concentration of 11% for spraying and moisturizing the corpses in the dissection lab (American Biosafety n.d.a.). First experiments were performed with corpses 16 and 17. Both were predissected by removing skin and subcutaneous adipose tissue and were treated by surface spraying of 11% InfuTrace™ and intrathoracic and intraabdominal injection of 20% InfuTrace™ (100 ml each). Only corpse 18 was re-perfused with InfuTrace™ .

The final method consisted of the application of InfuTrace™ to intact corpses fixed with embalming solution 1 one year before dissection. One week before the use of these corpses for dissecting purposes, they were pre-treated by surface spraying, multiple subcutaneous injections of 11% InfuTrace™ (3–5 l), and further intrathoracic (100 ml) and intraabdominal (100 ml) injections of 20% InfuTrace™.

To check the formaldehyde-binding effectiveness of the post-embalmment treatment with InfuTrace™ just before starting the dissection course, each corpse was tested using the directly displaying Draeger tubes: Formaldehyde 0,2a were used with the handheld accuro® pump (Drägerwerk AG & Co. KGaA, Lübeck, Germany).

For disinfection of corpses during their use in the dissection lab, Incidine® Liquid (Ecolab Deutschland GmbH) and FREKA®-NOL AF (Dr. Schumacher GmbH) were applied as spray.

## Results

Basic room levels of formaldehyde in the dissection hall ranged from 0.054 to 0.066 ppm, and in a side room connected to the main dissection lab without any air conditioning, the formaldehyde content was as low as 0.024–0.050 ppm.

### Decrease of formaldehyde values over the time the corpses are exposed to the air

**Fig. 3:**
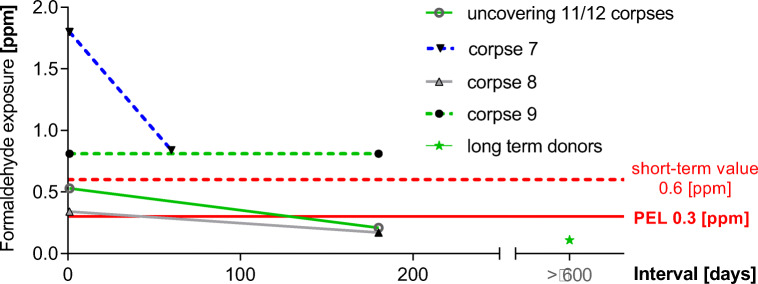
Time-dependent decrease of formaldehyde emission from corpses being exposed to the air for different time periods. The emissions distinctly decreased with the number of dissection units. Corpse 7, an evidently cachectic body, emitted high concentrations at the beginning of the dissection course (dissection of epifascial structures). Corpse 9, also obviously cachectic, did not show any reduction of formaldehyde emissions between 6 weeks and 32 weeks in use. The long-term donors displayed extremely low emissions of formaldehyde. Each corpse was fixed with 3% formaldehyde (solution 1). Measurements were performed without long throw nozzles, except for corpse 8

Samples were collected directly before the start of the dissection course during uncovering of the bodies. All bodies had been fixed with formaldehyde (solution 1). The collective of bodies (*n* = 12) being in use for 4 weeks was compared with the collective of bodies (*n* = 12) which had been in use for 7 months. The formaldehyde-fixed bodies had not been pre-treated in any way before measurement. The recorded average values were 0.53 ppm (4 weeks) and 0.22 ppm (7 months), respectively. The emission of formaldehyde dropped by about 55% (Fig. 3).

This observation was confirmed by further experiments. Repeated measurements of corpse 7 (evidently cachectic, no post-embalmment treatment) at two different points in time displayed formaldehyde emission values of 1.8 ppm (21 days after starting dissection) and 0.83 ppm 2 months later (Fig. 3, Table 1).

**Table 1:** Formaldehyde emission of one corpse during the dissection course measurements were performed with 2-month intermission (surface dissection and muscle dissection) **(**Fig. 3**)**

Corpse/dissection table	Day 1 (Corpse 3 weeks in use) Formaldehyde (ppm) ± SD	Two months later Formaldehyde (ppm) ± SD
Corpse 7 Table 10 without nozzles	1.8 ± 0.084	0.83 ± 0.084

Reduction of the formaldehyde emissions by the time the corpses were in use. The emissions clearly decrease with the number of dissection units (the time of corpse exposition in the dissection lab) (*n* = 5)

It is noteworthy that, in an additional experiment, the emissions of corpse 9, an evidently cachectic small body, did not decrease, with values of 0.82 ppm (first point in time) and 0.82 ppm 6 months later (second point in time) (Fig. 3 Table 2).

**Table 2:** Formaldehyde emissions of two corpses during the dissection course, measurements were performed with 6-month intermission in between (Fig. 3)

Corpse/dissection table	Day 1 (Corpses 3 months in use) Formaldehyde (ppm) ± SD	6 months later Formaldehyde (ppm) ± SD
Corpse 8 Table 1 with nozzles	0.35 ± 0.10	0.17 ± 0.0071
Corpse 9, cachectic Table 22 without nozzles	0.82 ± 0.11	0.82 ± 0.32

Corpse 9 was very cachectic and did not show any reduction. Each corpse was fixed with 3% formaldehyde (fixation 1). Measurements were performed without long throw nozzles, except for corpse 8. (*n*=16)

Long-term donors, in use for several years, when measured in a side room, displayed low formaldehyde emissions with values of 0.11 ppm in the breathing air, despite the fact that there was no ventilation.

Even when using long throw nozzles, there is an added reduction of emitted formaldehyde of 50% over time (corpse 8: 0.35 ppm first point in time down to 0.17 ppm 6 months later) (Fig. 3, Table 2).

### Formaldehyde emissions depend on the progress of dissection

**Fig. 4:**
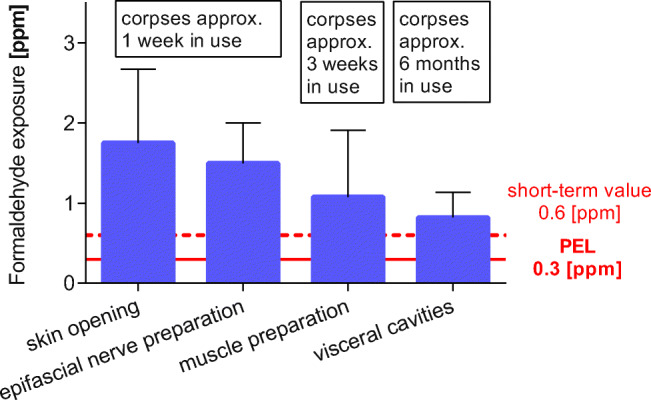
Formaldehyde emissions of corpses depend on the progress of dissection. Corpses fixed with 3% formaldehyde without post-embalming treatment in the dissection course. The emissions depend on the dissection progress and the time in use of the corpse. Skin and epifascial nerve dissection took place within the first 2 weeks. Corpses used for the dissection of muscles were 3 weeks in use, whereas those used for dissection of visceral cavities were in use for more than half a year (*n* = 18)

To test whether the emission of formaldehyde depends on the progress of dissection, the formaldehyde exposure was measured during the dissection course. Highest values were recorded during skin opening, with an average level of 1.8 ppm, followed by the removal of subcutaneous adipose tissue and dissection of epifascial nerves and vessels, with average levels of 1.5 ppm, and then 1.1 ppm during muscle dissection. Lowest formaldehyde concentrations were reached after opening of the inner cavities (0.82 ppm) after about 6 months (Fig. 4, Table 3).

**Table 3:** Formaldehyde emissions in relation to different dissection steps (Fig. 4)

Corpse/dissection table	Dissection step	Formaldehyde exposure (ppm) ± SD
Corpse 10 Table 1/2 Corpse 12 Table 1/2 Table 1 with and Table 2 without nozzles	Skin opening, New corpse	1.8 ± 0.92
Corpse 10 Table 1/2 Corpse 12 Table 1/2 Table 1 with and Table 2 without nozzles	Dissection of epifascial nerves New corpse	1.5 ± 0.50
Corpse 7 Table 10 without nozzles	Muscle dissection, Corpses 3 weeks longer in use	1.1 ± 0.83
Corpse 24 Table 1 with nozzles	Dissection of inner cavities, corpses 6 months longer in use	0.82 ± 0.32

Formaldehyde emissions of corpses fixed with 3% formaldehyde without post-embalming treatment. The emissions depend on the dissection progress and the time in use of the corpse. Skin and epifascial nerve dissection took place within the first 2 weeks. Corpses used for the dissection of muscles were 3 weeks in use, whereas those used for dissection of visceral cavities were in use for more than half a year. Measurements were performed without long throw nozzles, except for Table 1.. Data obtained at Table 1 were multiplied by the reduction factor 1.6 for means of comparison. (*n* = 18).

### Watering of the samples

Long-time watering of samples had no effect on the emitted formaldehyde. Measuring the emissions of the brain and spinal cord preparations displayed low emission values of 0.20–0.24 ppm, independent from the tissues being watered for several hours or not.

### Decrease in formaldehyde emissions by long throw nozzles

**Fig. 5:**
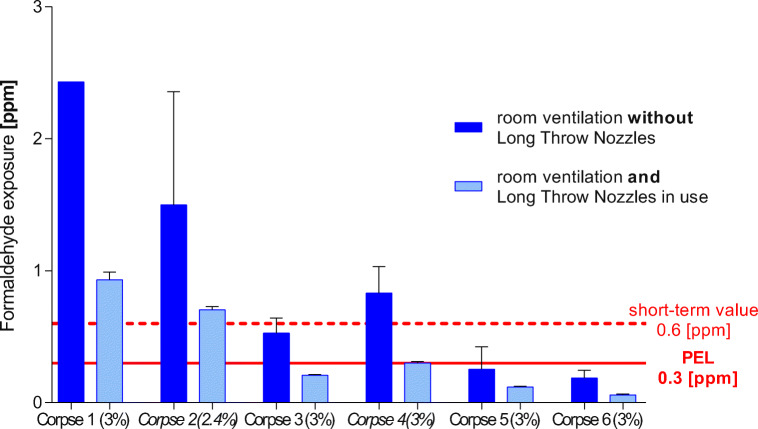
Decrease of formaldehyde by the use of long throw nozzles. The corpses 1 and 2 were recently fixed and measured without perfect adjustment of the nozzles; corpses 3 and 4 were used in the dissection courses for already half a year, corpse 5 was an obese body, and corpse 6 was treated with InfuTrace™

The formaldehyde exposure of students and teachers was measured using five different corpses during epifascial nerve preparation, except corpses 3+4 during muscle preparation, with and without the use of long throw nozzles (Fig. 5, Table 4). The results clearly revealed a strong reduction of formaldehyde exposure by more than 55% using exclusively the nozzle device. Yet, this does not guarantee adherence to the safety limit under all circumstances.

**Table 4:** Use of long throw nozzles vs. section hall ventilation without nozzles and without InfuTrace™ treatment (Fig. 5)

Corpse/formaldehyde in embalming fluid	Dissection step	Time in use* of corpses	Formaldehyde Exposure with nozzles (ppm)	Formaldehyde exposure without nozzles (ppm)	Formaldehyde reduction (%)
Corpse 1 3.0%	Epifascial nerve preparation	Fresh corpse	0.93 ± 0.056	1.5 ± 0.063	38
Corpse 2 2.4%	Epifascial nerve preparation	Fresh corpse	0.70 ± 0.0071	1.5 ± 0.86	55
Corpse 3 3.0%	Muscle preparation	6 months	0.21 ± 0.0071	0.53 ± 0.11	62
Corpse 4 3.0%	Muscle preparation	6 months	0.30 ± 0.014	0.83 ± 0.20	63
Corpse 5** 3.0%	Epifascial nerve preparation	Fresh corpse	0.12 ± 0.015	0.25 ± 0.17	50
Use of long throw nozzles vs. section hall ventilation without nozzles using InfuTrace™					
Corpse 6** 3.0%	Epifascial nerve preparation	Fresh corpse	0.058 ± 0.0078	0.18 ± 0.057	69

*in the dissection course

**obese or almost obese corpses

±SD standard deviation

The use of the long throw nozzles clearly causes a reduction of formaldehyde exposure a range of 38–69% (mean 56% ± 11%) with concentrations below the PEL. The long throw nozzles needed some optimization; therefore, the values of corpse 1 and 2 are still higher than the PEL. (*n* = 28)

### Emissions during skin opening of bodies fixed with embalming solutions containing reduced formaldehyde

**Fig. 6:**
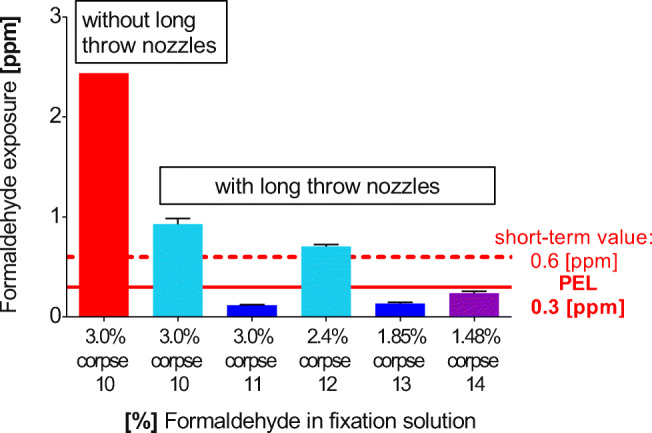
Formaldehyde emissions during skin opening depend on the formaldehyde content of the perfusion solution. Corpses were perfused either with 3% formaldehyde or with reduced formaldehyde in the perfusion solution. The samples were collected during a regular dissection course with additional ventilation by long throw nozzles. For comparison reasons, corpse 10 (red bar) was also measured without long throw nozzle ventilation. Cutting the formaldehyde in the embalming solution does not achieve compliance with the PEL. Corpse 10, 12, and 14: average weight; corpse 11 and 13: obese

For testing if lowering the formaldehyde concentration in the fixation can help to undercut the PEL requirements, various formulas for embalming solutions were tested during skin opening. Formaldehyde emission of a control (corpse 10: average-sized body donor, 3% formaldehyde fixation) without the use of long throw nozzles (2.4 ppm) was compared with measurements just 15 min later with long throw nozzles switched on (0.93 ppm). In contrast to the emissions of this average-sized body, an obese body (corpse 11; 3% formaldehyde, long throw nozzles switched on) revealed very low formaldehyde values of 0.12 ppm. Recordings of formaldehyde exposure around corpse 12 (average size, 2.4% formaldehyde) were 0.71 ppm, despite additional ventilation by long throw nozzles. The breathing air around the evidently obese corpse 13 (1.85% formaldehyde, long throw nozzle in use) contained 0.14 ppm formaldehyde, as compared with 0.24 ppm of the average-sized corpse 14 (salt solution, 1.48% formaldehyde, long throw nozzles in use) (Fig. 6, Table 5). Both obese corpses 11 and 13 were in the same low range indicating very low emission rates.

**Table 5:** Formaldehyde emissions of corpses fixed with low concentrations of formaldehyde, each scenario was measured with long throw nozzles in use, except the first scenario of the control corpse 10 (Fig. 6)

Corpse/dissection table	Dissection step	Formaldehyde exposure (ppm) ± SD
Corpse 10 Table 1 without long throw nozzles	3.0% formaldehyde (solution 1) Dissection of epifascial nerves	2.4
Corpse 10 Table 1	3.0% formaldehyde (solution 1) Dissection of epifascial nerves	0.93 ± 0.057
Corpse 11, obese Table 12	3.0% formaldehyde (solution 1) During skin opening, very obese corpse	0.12 ± 0.0058
Corpse 12 Table 1	2.4% formaldehyde (solution 2) During skin opening	0.71 ± 0.021
Corpse 13, obese Table 26	1.85% formaldehyde (solution 3) During skin opening	0.14 ± 0.013
Corpse 14 Table 25	1.48% formaldehyde (solution 4) During skin opening, salt-corpse	0.24 ± 0.017

Emissions of formaldehyde during skin opening at corpses with reduced formaldehyde in the perfusion solution. The samples were taken during a regular dissection course with installed and working long throw nozzles, except for the measurement of corpse 4 which was performed without long throw nozzles. The reduction of formaldehyde was not sufficient to keep emissions below the PEL for average weighing corpses. (*n* = 14)

The long throw nozzle system reduced exposure to formaldehyde by approximately 60% (Fig. 6, corpse 10). Emissions are well correlated to the formaldehyde concentration of the fixation solution if the corpses are of average size, cf. corpse 10 (embalming solution 2), corpse 12 (embalming solution 3), and corpse 14 (embalming solution 4) (*r* = 0.993). Two obviously obese corpses (11 and 13) displayed very low emission values, probably due to their high body mass index (BMI), and do not fit into the correlation.

**Fig. 7:**
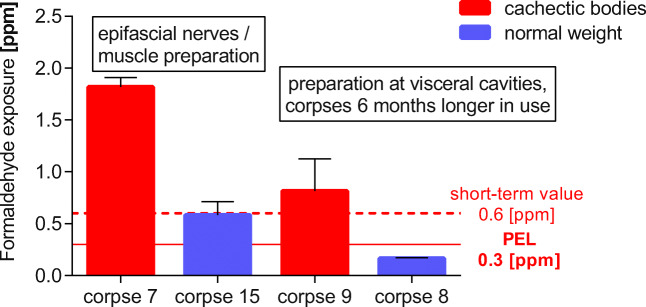
Formaldehyde exposure when using cachectic corpses (red) or average-sized corpses (blue), recorded during the same dissection session. Corpse 15 was measured below a long throw nozzle device. The measured values were therefore normalized by multiplication with a factor of 1.6 (60% reduction by the nozzles) to approximate the value for no additional directed airflow (i.e., without long throw nozzles)

It is noteworthy that corpses with very low weight showed much higher emissions than normal-sized corpses (Fig. 7, Table 6):

**Table 6:** Formaldehyde emissions of normal-sized corpses in comparison with cachectic corpses (Fig. 7)

Corpse/dissection table	Dissection step	Formaldehyde exposure (ppm) ± SD
Corpse 7, cachectic	Dissection of epifascial nerves	1.8 ± 0.092
Corpse 15	During skin opening	0.59 ± 0.13
Corpse 9, cachectic	Dissection of visceral cavities	0.82 ± 0.31
Corpse 8	Dissection of visceral cavities	0.17 ± 0.0071

Formaldehyde emissions of normal-sized corpses 15 and 8 in comparison with the obviously cachectic corpses 7 and 9; the dissection steps for each pair were similar; all corpses were fixed with 3% formaldehyde, no post-embalming treatment with InfuTrace™. Corpse 15 was originally measured below long throw nozzles in use, and therefore a factor of 1.6 basing on the 60% reduction was applied by the nozzles for means of comparison. (*n* = 17)

The cachectic corpse 7 showed formaldehyde emissions of 1.8 ppm during the dissection of epifascial nerves or muscles and corpse 15 (normal weight) of 0.59 ppm. Both corpses were in use for the same time period. Even if the corpses were in use for 6 months longer, cachectic corpses still emitted much more formaldehyde during comparable dissection steps: the cachectic corpse 9 emitted 0.82 ppm compared with 0.17 ppm from the average-sized corpse 8 (samples were collected during the dissection of visceral cavities (Fig. 7, Table 6).

Obviously, cachectic corpses emit higher amounts of formaldehyde than obese corpses.

### Post-embalmment treatment with InfuTrace™ and long throw nozzle effect

#### Thoracic and abdominal injection versus re-perfusion with InfuTrace™

All the following measurements were performed after additional ventilation was provided by the installation of the “three long throw nozzle system” over each dissection table.

All corpses were embalmed with solution 1. For comparison with corpse 10, which had not received InfuTrace™ treatment, two corpses (16 and 17, where the skin and adipose tissue had been removed, based on the hypothesis that these tissues emit high amounts of formaldehyde) were injected with 100 ml InfuTrace™ (20%) each into the thorax and the abdominal cavities (2 × 100 ml).

A third corpse 18, fixed 2 years before, was re-perfused with 20% of InfuTrace™. Re-perfusion was technically challenging due to the high counterpressure of the body during the process.

**Fig. 8:**
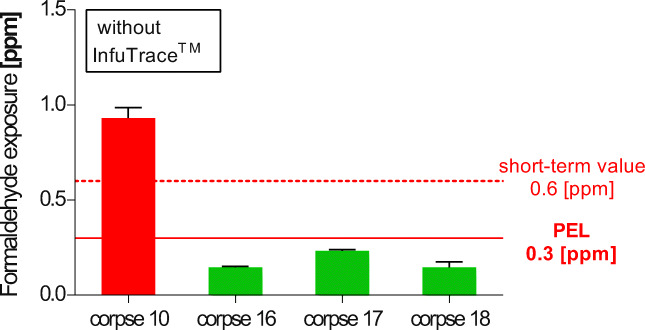
Effect of InfuTrace™: All corpses were positioned below long throw nozzles; corpse 10 without InfuTrace™ treatment, samples were taken during the dissection of the epifascial nerves (corpse 16 and 17 were without skin and without subcutaneous adipose tissue), cavities were injected with InfuTrace™, samples were taken during muscle dissection; corpse 18 had been re-perfused with InfuTrace™. Samples were taken during skin removal

Additionally, the surface of each corpse was treated with InfuTrace™ (11%) spray applied three times per week during the 2 weeks before sampling was performed. The InfuTrace™ treatment reduced formaldehyde emissions far below the German PEL and resulted in formaldehyde exposures of 0.15, 0.23, and 0.15 ppm, respectively, during dissection of muscles, fascia (corpses 16 and 17), and skin opening (corpse 18). The control corpse 10 without post-embalmment showed much higher values of 0.93 ppm during the dissection of the epifascial nerves (Fig. 8, Table 7).

**Table 7:** Treatment with InfuTrace™ of corpses fixed with 3% formaldehyde and using the long throw nozzles (Fig. 8)

Corpse/dissection table	Dissection step	Formaldehyde exposure (ppm) ± SD
Without InfuTrace™ treatment		
Corpse 10	Epifascial nerve dissection	0.93 ± 0.057
Without skin and sc. adipose tissue, InfuTrace™ treatment on body surface + visceral cavities		
Corpse 16	Dissection of muscles, fascia	0.15 ± 0.0058
Corpse 17	Dissection of muscles, fascia	0.23 ± 0.0058
Re-perfused with InfuTrace™, InfuTrace™ treatment on body surface		
Corpse 18	Skin opening	0.15 ± 0.029

All corpses were positioned below the long throw nozzles, corpse 10 without InfuTrace™ treatment, during epifascial nerve dissection; corpse 16 and 17 were without skin and without subcutaneous adipose tissue; cavities were injected with InfuTrace™; samples were taken during muscle dissection; corpse 18 was re-perfused with InfuTrace™: samples were taken during skin removal. (*n* = 9)

InfuTrace™ was also applied during the dissection process by fine spray distribution onto to the newly dissected areas of the corpses, to ensure a continuous binding of formaldehyde released during the dissection process. The combined procedure led to a reduction of formaldehyde exposure by about 80%.

However, using skinless corpses (predissected) does not allow the students to dissect skin, epifascial nerves, and veins and is thus unsatisfying for anatomical classes.

In the next step, the formaldehyde content in the embalmment solution was progressively reduced (embalmment solutions 4 to 6 utilized for corpses 19–22), and InfuTrace™ was applied as spray only on the surface of these whole bodies. Values from 0.13 to 0.22 ppm in the breathing air were reached during skin opening (Table 8).

**Table 8:** Emissions of corpses embalmed with formaldehyde-reduced perfusion and InfuTrace™ treatment applied as spray only on the surface of the corpses, using long throw nozzles

Corpse	Formaldehyde content of the fixation solution	Formaldehyde exposure (ppm) ± SD
Corpse 19	2.14% formaldehyde (solution 5)	0.18 ± 0.032
Corpse 20	2.14% formaldehyde (solution 5)	0.22 ± 0
Corpse 21	1.83% formaldehyde (solution 6)	0.18 ± 0.032
Corpse 22	1.48% formaldehyde (solution 4)	0.13 ± 0.0058

All corpses were positioned below the long throw nozzles and were embalmed with reduced content of formaldehyde. Each corpse was treated with InfuTrace™ only on its surface, without re-perfusion or injection into cavities. The samples were taken during skin removal. The formaldehyde exposure remained on a low level. (*n* = 11)

These InfuTrace™ applications appear to be suitable to meet the German PEL requirement of 0.3 ppm, but do not allow a lower PEL to be met, such as the Japanese one with 0.1 ppm, or the more stringent German requirements, as described below.

### Optimizing emissions by combining InfuTrace™ application with long throw nozzles

All the following measurements were performed after additional ventilation was provided by the installation of the “three long throw nozzle system” over each dissection table.

To lead through a complete dissection course beginning with skin opening and epifascial nerve dissection, while keeping formaldehyde emissions below the German “Stoffindex” value of 0.1 (“substance-index” - The German regulation for working places implies a so called “Stoffindex/substance-index below (<) 0.1, resp. 0.25” for the dissection practice (TRGS 402). Formula: measured value / (PEL x F). F = work shift/exposure time, for teachers F = 2.67 / for students F = 5.33 at the Anatomy in Giessen, Germany.), a method using 3% formaldehyde-fixed bodies for good preservation was amended by multiple additional subcutaneous injections of InfuTrace™ (11%) and injections into the visceral cavities (20%) 1 week before use in the dissection course. This application resulted in concentrations in the breathing air of 0.056 (corpse 23) and 0.036 ppm (corpse 24), respectively, during skin opening (Table 9).

**Table 9:** Emissions of corpses embalmed with a common 3% formaldehyde perfusion and InfuTrace™ treatment with 100 ml injections each into thorax, abdomen, and multi-subcutaneously

Corpse	Dissection step	Formaldehyde exposure (ppm) ± SD
Corpse 23	Skin opening	0.058 ± 0.0078
Corpse 24	Skin opening	0.036 ± 0.00058

Two corpses were embalmed with common 3% content of formaldehyde. Each corpse was treated with InfuTrace™ with 100 ml injections each into thorax, abdomen, and in addition multi-subcutaneously. The samples were taken during skin removal and using the long throw nozzles. The formaldehyde exposure fell far below 0.1 ppm. (*n* = 12)

**Fig. 9:**
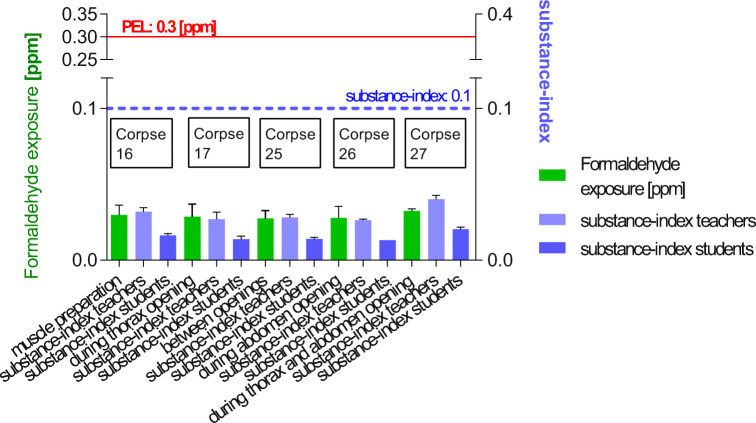
Formaldehyde exposure and substance-index registered during a regular dissection course: all corpses were pre-treated with InfuTrace™ by multiple-point injections and were exposed in the course for identical times. Samples were collected during opening of body cavities, either thorax and/or abdomen, single or simultaneously, where a high emission of formaldehyde was expected (long throw nozzles in use). All values stayed far below the German PEL and the required “substance-index” for teachers or for students

To corroborate this data, further samples were taken along the duration of a complete dissection course to cover all important steps in the advancing dissection progress. Critical dissection steps such as opening of the thorax and/or the abdomen with anticipated extremely high formaldehyde emissions were performed. The formaldehyde exposure (personal sampling) ranged from 0.021 to 0.036 ppm (Fig. 9, Table 10). The obtained values were far below the PEL and the German “Stoffindex” limits both for students and teachers (Fig. 9, Table 10).

**Table 10:** Formaldehyde exposure after successful reduction of formaldehyde emissions tested in a running dissection session with students, optimized InfuTrace™ treatment combined with long throw nozzles, corpses several months in use (Fig. 9)

Corpse/dissection table	Dissection step	Formaldehyde exposure (ppm)
Corpse 16 Table 26, cachectic dry corpse	Muscle dissection (0-15. min)	0.023 ± 0.0072
	During thorax opening (16.-30. min)	0.022 ± 0.0036
	Muscle dissection (31.-45. min)	0.023 ± 0.0015
	During abdomen opening (46.-60. min)	0.021 ± 0.00058
Corpse 17, Table 23, very obese corpse, very moisty	Muscle dissection (0-15. min)	0.026 ± 0.0065
	During thorax + abdomen opening (16.-30. min)	0.032 ± 0.0015
	Muscle dissection (31.-45. min)	0.028 ± 0.0057
Corpse 25 Table 24, corpse of common size	Muscle dissection (0-15. min)	0.034 ± 0.011
	During thorax opening (16.-30 min)	0.036 ± 0.010
Corpse 26 Table 18, corpse of common size	Muscle dissection (0-15. min)	0.031 ± 0.0040
	During abdomen opening (16.-30. min)	0.034 ± 0.0045
	Muscle dissection (31.-45. min)	0.031 ± 0.0040
Corpse 27 Table 17, corpse of common size	Muscle dissection (0-15. min)	0.034 ± 0.0052
	During thorax opening (16.-30. min)	0.030 ± 0.0032

The final experiment with corpses embalmed with common 3% content of formaldehyde: each corpse was treated with InfuTrace™ (100 ml injections each into thorax, abdomen, and in addition multi-subcutaneously). The samples were taken below the long throw nozzles during muscle dissection, during thorax opening, during abdomen opening, and—as a worst-case scenario—during simultaneously opening thorax and abdomen in a running students’ course. The formaldehyde exposure remained far below 0.1 ppm even in critical dissection steps. (*n* = 41)

### Use of different sampling methods

**Fig. 10:**
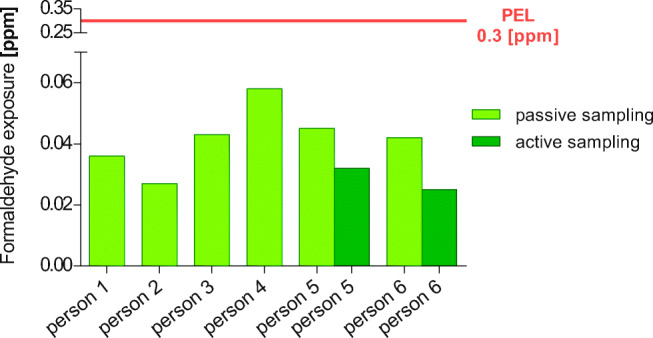
Formaldehyde exposure measured by passive sampler: teachers supervising two tables measured by passive sampling and active sampling. All values were far below the German PEL

Different sampling methods were used to test the reproducibility of the collected data, as well as to test the utility of passive samplers, which are less expensive and easier to handle. The test person can affix one by himself and send it to a lab for evaluation. Samples of the breathing air taken above corpses pre-treated with InfuTrace™ were collected both by a passive-sampling method (Radiello® passive sampler) and by active sampling (official BGIA-proceeding/NIOSH method 2016). The samplers were attached to teachers who moved between two dissection tables. The recorded values of both techniques excellently matched with concentrations of 0.045 ppm (passive sampler) and 0.032 ppm (active sampler) (person 5, dissection table 23 and 24) and with values of 0.042 ppm (passive sampler) and 0.025 ppm (active sampler) (person 6, dissection tables 17 and 18) (Fig. 10, Table 11).

**Table 11:** Formaldehyde exposure measured by Radiello® passive sampling versus active sampling, in a running dissection lab (Fig. 10)

Person*	Dissection table	Radiello® formaldehyde (ppm)	Active sampling formaldehyde (ppm)
Person 1	Table 25 + 26	0.036	–
Person 2	Table 27 + 28	0.027	–
Person 3	Table 21 + 22	0.043	–
Person 4	Table 20 + 19	0.058	–
Person 5	Table 23 + 24	0.045	0.032
Person 6	Table 17 + 18	0.042	0.025

*teachers are responsible for two dissection tables and moved between these tables

Formaldehyde exposure of teachers measured by passive and active sampling. All values were far below the German PEL and any “substance-index”. (*n* = 8)

### Reliability of the method

As a test for the reliability and efficacy of the described method for ensuring extremely low formaldehyde exposure values, measurements were performed under extreme climate conditions with outside temperatures of 38 °C and a relative air humidity of 85% inside the dissection hall. The concentrations of formaldehyde exposure and of the ambient air increased with rising temperatures in the dissection hall (up to 20 °C) by about 40–60% but still remained far below the PEL value and below the German “Stoffindex,” both for students and teachers (Table 12).

**Table 12:** Left, formaldehyde exposure measured at common climate conditions (17 °C/33% r.h.*); right, formaldehyde exposure measured at a very hot summer day (20 °C/78% r.h.*) 7 weeks later

Corpse/dissection table	Formaldehyde (ppm) ± SD 17.7 °C/33% r.h.	Formaldehyde (ppm) ± SD 20 °C/78% r.h.
Corpse 26, Table 18	0.023 ± 0.0028	0.056 ± 0.0078
Corpse 7, Table 26, cachectic little corpse	0.032 ± 0.0040	0.052 ± 0.0066

*room temperature/relative humidity r.h

Formaldehyde exposure of teachers increased by about 40–60% with the high room temperature and extremely high relative humidity, but the values still remained far below the German PEL and any “substance-index”. (*n* = 27)

For controlling the InfuTrace™ effect in freshly treated corpses (1 week after the first post-embalming treatment with InfuTrace™), Draeger gas tubes were used. The samples were collected during the first skin opening. The registered values stayed all below the detection limit of 0.2 ppm each and corresponded to the later determined low values during the dissection course.

Most importantly, the applied methods were validated by the Regierungspräsidium Kassel, a governmental measuring authority. In a first validation step, samples were collected during a dissection course, where the skin was dissected from corpses fixed with 3% formaldehyde and post-embalmment InfuTrace™ treatment and a corpse also fixed with 3% formaldehyde but without further treatment. All previously registered data was verified. None of the InfuTrace™-treated corpses 28 and 29 led to formaldehyde concentrations in the breathing air above 0.06 ppm (maximum). The samples of the control corpse 30 without InfuTrace™ treatment displayed a concentration of 0.33 ppm being close to the PEL. Compared with the post-embalmment-treated corpses (Table 13) with long throw nozzles running, the efficacy of the post-embalmment method was confirmed with these low exposure rates.

**Table 13:** Sampling and chemical analysis by the Regierungspräsidium Kassel of corpses which were in use for half a year and with optimized InfuTrace™ treatment and control corpse 30 without InfuTrace™ treatment and nozzles in work

Corpse/dissection table	Dissection procedure	Formaldehyde exposure (ppm) ± SD
Corpse 28, Table 1	Skin dissection	0.038 ± 0,0068
Corpse 29, Table 2	Dissection of muscles	0.057 ± 0.019
Control corpse 30, Table 11	Skin dissection	0.24 ± 0.084

Measurements by the governmental authority during skin dissection and dissection of muscles using long throw nozzles on corpses treated with InfuTrace™ that were half a year in use confirmed the low formaldehyde exposures with results below 0.1 ppm. The control corpse 30 without InfuTrace™- treatment resulted in much higher concentrations up to 0.32 ppm. (*n* = 21)

In addition, control measurements by the Regierungspräsidium Kassel were also performed during the opening of the abdomen of corpses having been in use for about half a year in a second-term course. They corroborated all previous data with values between 0.019 and 0.036 ppm (Table 14).

**Table 14:** Sampling and chemical analysis by the Regierungspräsidium Kassel of corpses which were several months in use and with optimized InfuTrace™ treatment and nozzles in work

Corpse/dissection table	Dissection procedure	Formaldehyde exposure (ppm) ± SD
Corpse 24 + 31, Table 1 + 2	Opening and dissection of the abdomen	0.019 ± 0.010
Corpse 23 + 32, Table 7 + 8	Opening and dissection of the abdomen	0.036 ± 0,0084
Corpse 11+ 33, Table 9 + 10	Opening and dissection of the abdomen	0.019 ± 0.0063
Corpse 29 + 34, Table 11 + 12	Opening and dissection of the abdomen	0.021 ± 0.0077

Measurements by the governmental authority during opening and dissection of the abdomen using long throw nozzles on corpses treated with InfuTrace™ and several months in use confirmed the low formaldehyde exposures with results far below 0.1 ppm. Samples were taken at teachers who are responsible for two dissection tables and moved between these tables. (*n* = 22).

The procedure was repeated in the beginning of a first-term student course, and the low formaldehyde emission values were confirmed. Corpses which had been dissected just 1 or 2 weeks after post-embalmment partly displayed slightly higher emission rates ranging from 0.046 ppm to 0.089 ppm (Table 15), as compared with samples taken from corpses in the previous second-term course.

**Table 15:** Sampling and chemical analysis by Regierungspräsidium Kassel, corpses have been a few weeks in use and with optimized InfuTrace™ treatment and working nozzles

Corpse/dissection table	Dissection procedure	Formaldehyde exposure (ppm) ± SD
Corpse 35 + 36, Table 3 + 4	Skin dissection and 7 dissection of fat tissue	0.056 ± 0.00071
Corpse 36, Table 4	Skin dissection and dissection of fat tissue	0.053 ± 0,0064
Corpse 37 + 38, Table 9 + 10	Skin dissection and dissection of fat tissue	0.046 ± 0.00071
Corpse 39, Table 11	Skin dissection and dissection of fat tissue	0.076 ± 0.0028
Corpse 40, Table 12	Skin dissection and dissection of fat tissue	0.089 ± 0.011

Measurements by the governmental authority in a students’ course (skin dissection and dissection of fat tissue) using long throw nozzles on corpses treated with InfuTrace™ and only a few weeks in use confirmed the low formaldehyde exposures with results below 0.1 ppm. Samples were taken at teachers who are responsible for two dissection tables and moved between these tables. (*n* = 12)

In summary, the combination of the long throw nozzle system and post-embalmment treatment with InfuTrace™ is a very solid, efficient, and reliable strategy for reducing formaldehyde emissions from 3% formaldehyde-fixed corpses.

## Discussion

### The formaldehyde emission process

The process of formaldehyde emission from embalmed corpses follows the laws of thermodynamics and is affected by various factors: concentration of formaldehyde in the fixation, the applied amount of fixating-solution, formaldehyde-content of the corpse (Thullner et al. 2015), the body mass index (BMI), the time the corpse is exposed to air, dissection methods and the progress in dissection, number of persons surrounding the corpse, ventilation of the dissection classroom as well as of the dissection table, and exterior climate (temperature and relative air humidity). Thus, the data concerning formaldehyde exposure in dissection classes reported in literature have to be evaluated carefully.

Highest formaldehyde concentrations were measured at the beginning of the dissection course when the skin is removed and muscles are dissected. Within a few weeks, the formaldehyde emission decreased by up to 60%. Long-time donors used for exams fixed with 3% formaldehyde displayed very low formaldehyde emissions. Our data were corroborated by Perkins and Kimbrough (1985) and Shiraishi (2006), who reported a 50% decrease of formaldehyde emission.

A second peak of formaldehyde emission is expected during the opening of body cavities. As seen in our experiments, the absolute emission values are lower, compared with skin dissection, corresponding to the time of the cadavers being in use in the course (6 months)**.** Shiraishi (2006) also reported highest values of formaldehyde emission either during skin opening or when dissecting the viscera.

It is supposed that adipose tissue releases more formaldehyde compared with other tissues. This was not confirmed in this study. Values from obese bodies displayed very low concentrations of emitted formaldehyde in comparison with normal weight corpses. By contrast, obviously cachectic bodies displayed very high levels of formaldehyde. It is noteworthy that these high concentrations of formaldehyde emission persisted over time.

The skin and the adipose tissue build a barrier for the emission of formaldehyde, as formaldehyde is insoluble in fat. Brains and nervous tissue without any pre-treatment to reduce formaldehyde emitted very low quantities of formaldehyde. It is noteworthy that rinsing brains for several hours with water did not affect the formaldehyde emission. As the nervous tissue contains high concentrations of lipids (70%) (Labadie and Möller 2010), low emission values can be attributed to the insolubility of formaldehyde in lipids. These data are corroborated by Sugata et al. (2016) reporting lower emissions from adipose tissue (0.4 ppm) compared with that of muscle tissue (0.9 ppm). Thus, the hypothesis that adipose tissue emits high amounts of formaldehyde has to be revised.

### Reduction of formaldehyde content in perfusion solution

The presented project aimed to develop a technique to undercut any governmental limitation of formaldehyde exposure in dissection rooms. One possible approach to lower the exposure to formaldehyde emitted from fixed corpses is to lower the concentration of formaldehyde in the fixation (Thullner et al. 2015). Yet this approach, using concentrations as low as 1.5%, did not achieve the expected results for undercutting the PEL. Further reduction of the amount of applied formaldehyde endangers the quality of fixation and increases the risk for infections (Spaethe 2003). In our study, a concentration of 3% formaldehyde with about 20 L perfusion solution for each cadaver is sufficient for excellent fixation results, but it should, for reasons of fixation and safety, not be lowered any further.

### Reduction of formaldehyde exposure by means of ventilation

Up to now, expensive and space-consuming solutions by extraction tables requiring a costly infrastructure seemed to be a real promising strategy. Different models of extraction tables relying on varying air suction systems are on the market (Klein et al. 2014; Kunugita et al. 2004). Some of these extraction tables are capable of reducing the formaldehyde exposure below the PEL (Coleman 1995; Kikuta et al. 2010; Shinoda and Oba 2010; Yamato et al. 2005). The newest development is an extraction table using turbinal aspiration of the formaldehyde loaded air for its effective removal (ROM system; Rudolf Otto Meyer Technik, Stuttgart, Germany) (Stockmann 2016), which needs spacious technology. An alternative approach to reducing the formaldehyde concentration in ambient and breathing air is a local ventilation system of the pull-push type (Matsuda et al. 2009).

Restrictions imposed by the constructional preconditions of our Anatomical department prohibited the use of such devices. By developing a different built-in system — the use of the long throw nozzles attached to the ceiling over each dissection table—this obstacle was circumvented. The formaldehyde exposure to emissions from conventionally 3% formaldehyde-fixed bodies was reduced by more than 55%, thus almost meeting the PEL requisitions. The guided airflow prevented the thermally induced updraft of contaminated air by directing it to the ground where it was exhausted. Such a long throw nozzle device can be installed in and adapted to any human or veterinary gross anatomy laboratory. It is noteworthy that this is extraordinary cost effective. The cost to equip our 12 tables in the dissection room amounted to about € 80,000. Yet, the costs may vary depending on the type of nozzle-system needed, taxes, transport, and other.

### Chemical options for further reduction of formaldehyde exposure

The German regulation for working places demands compliance with a so-called substance-index. In consequence, values below 0.08 ppm for teachers had to be reached in the Anatomical Department of Giessen to attest “protective measures adequate” for safe working according to the “technical rules for hazardous substances” (Ausschuss für Gefahrstoffe 2010).

To meet the substance-index requirement, in addition to the PEL, an formaldehyde-destroying agent was applied. Coskey and Gest (2015) and Burkel et al. (1999) described monoethanolamine (MEA) — it produces an imine alcohol with formaldehyde — which was re-perfused and injected at multiple points in 6% formaldehyde-fixed corpses, thus reaching formaldehyde exposure values of 0.41 ppm. Alternatively, a saturated ammonium carbonate solution was used for re-perfusion of corpses that were fixed with 3.7% formaldehyde by Kawamata and Kodera (2004). Formaldehyde is captured to build hexamethylenetetramine. This approach resulted in formaldehyde concentrations of 0.2 to 1.0 ppm. Frölich et al. (1984) and Becker (2003) applied phenoxyethanol to corpses fixed with a solution of 2–4% formaldehyde, which resulted in concentrations below 0.5 ppm. The method is laborious and expensive. A completely different way for formaldehyde destruction is photocatalysis as used by Ohmichi et al. (2006). A reduction in vapors of 70–80% was achieved and resulted in concentrations of 0.1–0.95 ppm. But the method is intricated.

Due to the drawbacks of these methods, we tested InfuTrace™ post-embalmment treatment. Cauwenbergs et al. (n.d.) first described InfuTrace™, a solution reducing free formaldehyde and phenol by polymerization in fixed corpses with an efficiency of up to 90%. The diluted solution can be applied by re-perfusion, injections into the visceral cavities, as well as a surface spray. Criticism came from Burkel et al. (1999), who complained about precipitates on tissues and on dissection tables. Demer cites Sleek and coworkers in an e-mail, who showed “significant decreases of formaldehyde levels between 42 to 84% through the use of InfuTrace™ treated cadavers when compared with their untreated counterparts using 5% formaldehyde” (Demer 2014). Nevertheless, quite a lot of American universities apply InfuTrace™ in dissection courses (Daemen College, (Styn 2014–2015), Mt. San Antonio College (2014), Western Carolina University (Caler et al. 2011), and others). Whitehead and Savoia (2008) compared post-embalming InfuTrace™ treatment of 2% formaldehyde-fixed corpses by injection into cavities or re-perfused corpses. Slightly higher values were seen after re-perfusion with InfuTrace™. Goldman (2010) achieved “dramatic” and Cope et al. (2009) significant reductions of formaldehyde concentrations in the air after re-perfusion with InfuTrace™, but according to Goldman, the growth of mold increased. Up to now, solid proof for the effectiveness of InfuTrace™ for achieving German working place standards with a very low PEL was lacking. Despite reports on extraordinarily good results and experiences using InfuTrace™, this formula seems to be quite unknown in Europe and further countries all over the world.

In our setting, the application of InfuTrace™ by multiple-point subcutaneous injections resulted in a 60% reduction of formaldehyde exposure. Treating 24 corpses costs about € 1000 per year (including tax and shipping). Up to now, the chemical ingredients are unknown. Yet, the mechanism of using urea to build formaldehyde polymers in resins is widely used. A comparable mechanism might be the basis of InfuTrace™ effects.

Precipitates can be eliminated with water. Adding glycerin to the InfuTrace™-spray solution prevents both the drying of corpses and the development of precipitates. In case of mold growth, Incidine® liquid can be used as wetting solution to efficiently prevent mold growth.

Differences evoked by varying BMI and dissection procedures were reduced by the presented combination of a long throw nozzle system for airflow direction and InfuTrace™ polymerizing free formaldehyde. The achieved reduction of formaldehyde air pollution in the dissection labs undercuts any given limit.

### Long-term monitoring of formaldehyde exposure

These low concentrations allow recordings of formaldehyde emissions using the Radiello® passive-sampler system. The correlation with active sampling was excellent. Yim et al. (2013) found good correlations with a similar passive-sampler method based on DNPH as adsorbent. Due to the lower position of the passive sampler, closer to the emission source — the corpses — the passive sampler achieved slightly higher values in our experiments, compared with those seen by the active sampling method. The passive sampler offers a new possibility to use it like an individual dosimeter, as it can be applied in each dissection unit along the complete duration of the dissection course. Thus, the passive sampler can record the average concentration of formaldehyde the individual had been exposed to. This method to control the PEL might be useful for effectively tracing irregular emission processes arising during dissection courses. This will provide additional valuable information about long-term formaldehyde exposure.

## Conclusion

Our results present an efficient and cost-effective possibility for keeping the formaldehyde concentration of the air in dissection classes below critical values, using both improved ventilation and a method to polymerize free formaldehyde by InfuTrace™. The combined method allowed reaching values of formaldehyde concentrations in the ambient air well below the German PEL of 0.3 ppm and even below 0.08 ppm. The formaldehyde concentration in the ambient air of the dissection class room decreased by 90%, with 0.21 ppm before and 0.019 ppm afterward.

**Fig. 11:**
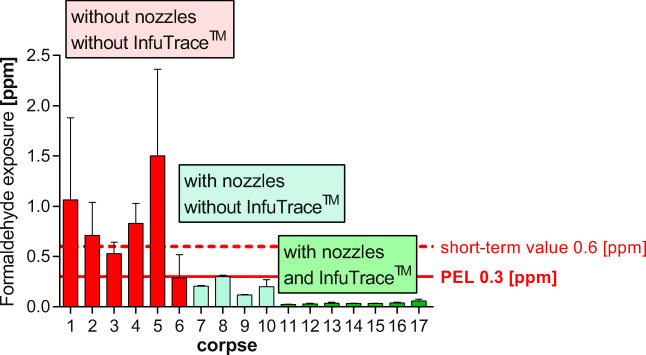
Graphical summary. Steps of formaldehyde reduction: red bars before any reduction methods were introduced, the PEL could not be fulfilled; blue bars using long throw nozzles, the PEL was fulfilled; green bars using long throw nozzles and InfuTrace™ by multiple-point injection with values far below the PEL (Appendix Table 16)

The application of InfuTrace™ is easy and contributes to safe working for teachers and students. The exclusive use of InfuTrace™ reduces formaldehyde emissions by about 70%. The newly developed three long throw nozzle systems can be adapted and installed in any dissection room (adding nozzles per table or increasing the directed airflow rate). The sole application of the long throw nozzles reduced the formaldehyde exposure by 60%. Combining both methods resulted in a reduction of about 90% (graphical summary Fig. 11, Table 16). The installation of suction tables including all necessary devices in the dissection hall and in the background is much more costly; thus, our method is not only efficient but also very cost-effective.

**Table 16:** Graphical summary formaldehyde emissions before formaldehyde reduction in a students’ dissection course without long throw nozzles and without InfuTrace™ treatment (Fig. 11, graphical summary)

Corpse	Dissection step	Formaldehyde exposure (ppm) ± SD
Corpse 1	Epifascial nerve dissection	1.1 0.82
Corpse 2	Inner cavities	0.71 ± 0.33
Corpse 3	Inner cavities	0.53 ± 0.11
Corpse 4	Inner cavities	0.83 ± 0.20
Corpse 5	Epifascial nerve dissection	1.5 ± 0.86
Corpse 6	Skin opening	0.28 ± 0.17
Formaldehyde emissions were measured with long throw nozzles in use, without InfuTrace™ treatment		
Corpse 7	Inner cavities	0.21 ± 0.0071
Corpse 8	Inner cavities	0.30 ± 0.014
Corpse 9	Muscles	0.12 ± 0.00058
Corpse 10	Muscles	0.20 ± 0.070
Formaldehyde emissions after successful reduction of formaldehyde emission tested in a running dissection session with attending students and optimized InfuTrace™ treatment combined with long throw nozzles		
Corpse 11	Inner cavities	0.023 ± 0.0028
Corpse 12	Inner cavities	0.029 ± 0.0053
Corpse 13	Inner cavities	0.035 ± 0.0096
Corpse 14	Inner cavities	0.032 ± 0.0040
Corpse 15	Inner cavities	0.031 ± 0.0041
Corpse 16	Skin dissection	0.038 ± 0.0065
Corpse 17	Muscles	0.057 ± 0.019

Graphical summary: corpses 1–6 without InfuTrace™ treatment and without long throw nozzles in use; corpses 7–10 without InfuTrace™ treatment and with long throw nozzles in use; corpses 11–17 with optimized InfuTrace™ treatment and with long throw nozzles in use. Starting with formaldehyde exposures up to 1.5 ± 0.86 ppm the exposure finally did not exceed 0.057 ± 0.019 ppm. (Corpses 1–6 *n* = 32; corpses 7–10: *n* = 10, corpses 11–17: *n* = 59)

To sum up, the combined method of a modified easy-to-install air ventilation — three long throw nozzles positioned at the ceiling along the longitudinal axis of the dissection table — and the post-embalmment treatment with InfuTrace™ complies with all known PEL limits, even as low as the Japanese, with values of 0.1 ppm, for formaldehyde exposure in dissection classes over the time of a complete dissection course. Even worst-case scenarios such as higher temperature or extreme air humidity in the dissection lab did result neither in higher concentrations of formaldehyde in the ambient air nor in higher exposure to formaldehyde. Thus, the reproducibility of these results corroborates the validity of the method, which might contribute to the reduction of formaldehyde exposure in dissection labs all over the world.

## Electronic supplementary material

ESM 1 (PDF 237 kb).
