# Supplementary material for: Low cost and effective reduction of formaldehyde in gross anatomy: long throw nozzles and formaldehyde destruction using InfuTrace™
Source: Environ Sci Pollut Res Int. 2020 Aug 11;27(36):45189–208. doi: 10.1007/s11356-020-09961-0 (PMC7686167; doi:10.1007/s11356-020-09961-0)
Supplement: Supplementary file 1 — (PDF 237 kb). [file 11356_2020_9961_MOESM1_ESM.pdf]

Low Cost and Effective Reduction of Formaldehyde in Gross Anatomy: Long Throw Nozzles and Formaldehyde Destruction using INFUTRACE^{TM}

Environmental Science Pollution Research

Sonja Pfeil[1*], Hans Hieke[2], Petra Brohmann[3], Monika Wimmer[1,4]

[1]*Institute of Anatomy and Cell Biology, Justus-Liebig-University, Aulweg 123, 35392 Giessen, Germany*

[2]*Department of Real Estate, Construction and Technology, Justus-Liebig-University, Ludwigstraße 23, 35390 Gießen, Germany*

[3]*Department 35.3, Kassel Regional Council, Ludwig-Mond-Str. 33, 34121 Kassel, Germany*

[4]*Institute of Anatomy Johannes Kepler University Linz, Huemerstraße 3-5, 4020 Linz, Austria*

Sonja.Pfeil@anatomie.med.uni-giessen.de

Appendix

Embalming solution 2 -6, ingredients

Embalming solution 2: **2.4%** formaldehyde, 5% phenoxyethanol, 5% glycerin, 64% ethanol, deionized water.

*Embalming solution 3:***1.85%** formaldehyde, 0.83% thymol, 0.83% glycerin, 0.83% lysoformin, 0.83% diethylenglycol, 21.7% ethanol, deionized water.

Embalming solution 4: saturated salt solution (NaCl) with **1.48%** formaldehyde, 2% glycerin, 16% ethanol, 10 kg sodium chloride, deionized water.

Embalming solution 5: **2.14%** formaldehyde, 2.5% phenoxyethanol, 2.5% glycerin, 87% ethanol, deionized water.

Embalming solution 6: **1.83%** formaldehyde, 0.83% phenol, 0.83% thymol, 0.83% glycerin, 0.83% lysoformin, 0.83% diethylenglycol, 21.7% ethanol, deionized water.

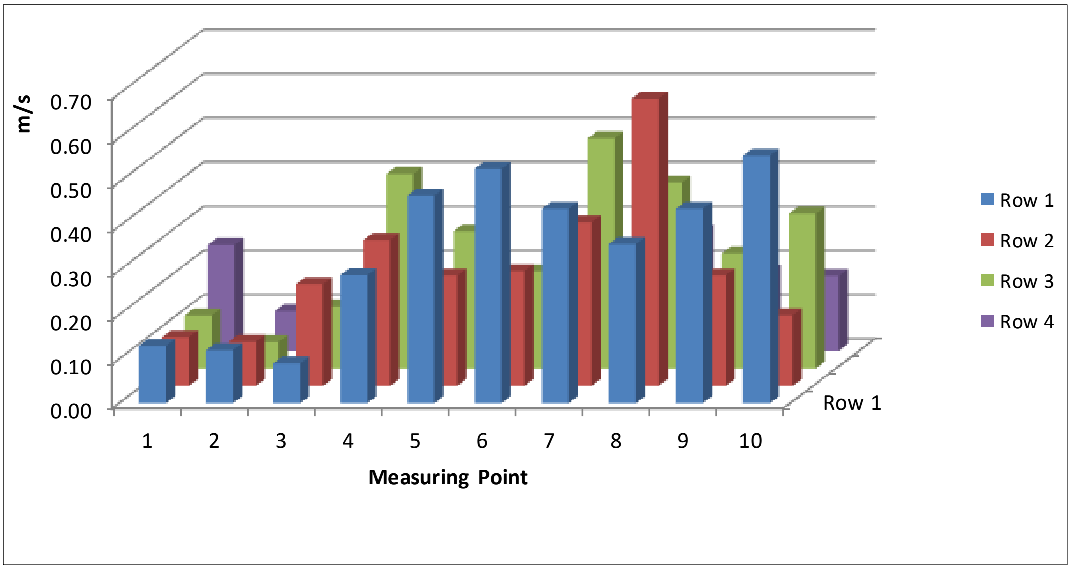

Fig. 2 Airflow with running long-throw-nozzles measured approx. 20 cm above the cadaver placed on the dissection table with highest velocity above the thorax and the low velocity above the head and legs. The additional air jet does not affect comfortable working at the table

**Table 1**: **Formaldehyde emissions of corpse 1 during the dissection course measurements were performed with two months intermission** (surface-dissection and muscle dissection) **(Fig. 3)**		
Corpse / dissection table	*day 1 (corpse 3 weeks in use) formaldehyde (ppm) ± SD*	*two months later formaldehyde (ppm) ± SD*
Corpse 7<br>Table 10 without nozzles	1.8 ± 0.084	0.83 ± 0.084
**Table 2: Formaldehyde emissions of two corpses during the dissection course, measurements were**		
Corpse / dissection table	*day 1 (corpses 3 Months in use) formaldehyde (ppm) ± SD*	*6 months later formaldehyde (ppm) ± SD*
Corpse 8<br>Table 1, with nozzles	0.35 ± 0.10	0.17 ± 0.0071
Corpse 9, cachectic<br>Table 22 without nozzles	0.82 ± 0.11	0.82 ± 0.32

Table 1+2: Decrease of the formaldehyde exposure by the time the corpses were in use. The emissions clearly decrease with the number of dissection units (the time of corpse exposition in the dissection lab). Corpse 9 was very cachectic and did not show any reduction. Each corpse was fixed with 3% formaldehyde (fixation 1). Measurements were performed without long throw nozzles, except for corpse 8. (Corpse 7: n = 5; Corpse 8+9: n=16)

Table 3: Formaldehyde emissions in relation to different dissection steps (Fig. 4)		
Corpse / dissection table	*Dissection step*	*Formaldehyde exposure(ppm) ± SD*
Corpse 10 Table 1/2 Corpse 12Table 1/2<br>Table 1 with and Table 2 without nozzles	skin opening, new corpse	1.8 ± 0.92
Corpse 10 Table 1/2 Corpse 12 Table 1/2<br>Table 1 with and Table 2 without nozzles	dissection of epifascial nerves, new corpse	1.5 ± 0.50
Corpse 7<br>Table 10 without nozzles	muscle dissection, corpses 3 weeks longer in use	1.1 ± 0.83
Corpse 24<br>Table 1 with nozzles	dissection of inner cavities, corpses 6 months longer in use	0.82 ± 0.32

Table 3: Formaldehyde emissions of corpses fixed with 3 % formaldehyde without post-embalming treatment. The emissions depend on the dissection progress and the time in use of the corpses. Skin and epifascial nerve dissection took place within the first two weeks. Corpses used for the dissection of muscles were three weeks in use, whereas those used for dissection of visceral cavities were in use for more than half a year. Measurements were performed without long throw nozzles, except for table 1. Data obtained at table 1 were multiplied by the reduction factor 1.6 for means of comparison. (n = 18)

Table 4: Use of long-throw nozzles vs. section hall ventilation without nozzles and without InfuTrace™-treatment (Fig. 5)					
Corpse / Formaldehyde in embalming fluid	*Dissection step*	*Time in use* of corpses*	*Formaldehyde Exposure with nozzles (ppm)*	*Formaldehyde Exposure without nozzles (ppm)*	*Formaldehyde reduction (%)*
Corpse 1<br>3.0%	Epifascial nerve preparation	Fresh corpse	0.93 ± 0.056	1.5 ± 0.063	38
corpse 2<br>2.4%	Epifascial nerve preparation	Fresh corpse	0.70 ± 0.0071	1.5 ± 0.86	55
Corpse 3<br>3.0%	Muscle preparation	6 months	0.21 ± 0.0071	0.53 ± 0.11	62
Corpse 4<br>3.0%	Muscle preparation	6 months	0.30 ± 0.014	0.83 ± 0.20	63
Corpse 5**<br>3.0%	Epifascial nerve preparation	Fresh corpse	0.12 ± 0.015	0.25 ± 0.17	50
**Use of long-throw nozzles vs. section hall ventilation without nozzles using InfuTrace™**					
Corpse 6**<br>3.0%	Epifascial nerve preparation	Fresh corpse	0.058 ± 0.0078	0.18 ± 0.057	69

*: in the dissection course **: obese or almost obese corpses ± SD: standard deviation

Table 4: The use of the long throw nozzles clearly causes a reduction of formaldehyde exposure in a range of 38-69% (mean 56% ± 11%) with concentrations below the PEL. The long throw nozzles needed some optimization, therefore the values of corpse 1 and 2 are still higher than the PEL. (n=28)

**Table 5: Formaldehyde emissions of corpses fixed with low concentrations of formaldehyde**, each scenario was measured with long-throw nozzles in use, except the first scenario of the control corpse 10 **(Fig. 6)**		
Corpse / dissection table	*Dissection step*	*Formaldehyde exposure (ppm) ± SD*
Corpse 10<br>Table 1 without long throw nozzles	*3.0% formaldehyde (solution 1)*<br>Dissection of epifascial nerves	2.4
Corpse 10<br>Table 1	*3.0% formaldehyde (solution 1)*<br>Dissection of epifascial nerves	0.93 ± 0.057
Corpse 11, obese<br>Table 12	*3.0% formaldehyde (solution 1)*<br>During skin opening, very obese corpse	0.12 ± 0.0058
Corpse 12<br>Table 1	*2,4% formaldehyde (solution 2)*<br>During skin opening	0.71 ± 0.021
Corpse 13, obese<br>Table 26	*1,85% formaldehyde (solution 3)*<br>During skin opening,	0.14 ± 0.013
Corpse 14<br>Table 25	*1,48% formaldehyde (solution 4)*<br>During skin opening, salt-corpse	0.24 ± 0.017

Table 5: Emissions of formaldehyde during skin opening at corpses with reduced formaldehyde in the perfusion solution. The samples were taken during a regular dissection course with installed and working long throw nozzles, except for the measurement of corpse 4 which was performed without long throw nozzles. The reduction of formaldehyde was not sufficient to keep emissions below the PEL for corpses with average weight. (n=14)

**Table 6: Formaldehyde emissions of normal sized corpses in comparison to cachectic corpses (Fig. 7)**		
Corpse / dissection table	*Dissection step*	*Formaldehyde exposure (ppm) ± SD*
Corpse 7, cachectic	Dissection of epifascial nerves	1.8 ± 0.092
Corpse 15	During skin opening	0.59 ± 0.13
Corpse 9, cachectic	Dissection of visceral cavities	0.82 ± 0.31
Corpse 8	Dissection of visceral cavities	0.17 ± 0.0071

Table 6: Formaldehyde emissions of normal sized corpses 15 and 8 in comparison to the obviously cachectic corpses 7 and 9, the dissection steps for each pair were similar, all corpses were fixed with 3 % formaldehyde, no post-embalming treatment with InfuTrace^{TM}. Corpse 15 was originally measured below long throw nozzles in use and therefore a factor of 1.6 basing on the 60% reduction was applied by the nozzles for means of comparison. (n = 17)

**Table 7: Treatment with InfuTrace™ of corpses fixed with 3% formaldehyde and using the long throw nozzles (Fig. 8)**		
Corpse / dissection table	*Dissection step*	*Formaldehyde exposure (ppm) ± SD*
without InfuTrace™ treatment		
Corpse 10	Epifascial nerve dissection	0.93 ± 0.057
without skin and sc. adipose tissue, InfuTraceTM treatment on body-surface + visceral cavities		
Corpse 16	Dissection of muscles, fascia	0.15 ± 0.0058
Corpse 17	Dissection of muscles, fascia	0.23 ± 0.0058
Re-perfused with InfuTrace™, InfuTrace™ treatment on body-surface		
Corpse 18	Skin opening	0.15 ± 0.029

Table 7: All corpses were positioned below the long throw nozzles, corpse 10 without InfuTrace™-treatment, during epifascial nerve dissection; corpse 16 and 17 were without skin and without subcutaneous adipose tissue, cavities were injected with InfuTrace™, samples were taken during muscle dissection; corpse 18 was re-perfused with InfuTrace™: samples were taken during skin removal. (n=9)

**Table 8: Emissions of corpses embalmed with formaldehyde reduced perfusion and InfuTrace™-treatment** applied as spray only on the surface of the corpses, using long throw nozzles		
Corpse	*Formaldehyde content of the fixation solution*	*Formaldehyde exposure (ppm) ± SD*
Corpse 19	2.14 % formaldehyde (solution 5)	0.18 ± 0.032
Corpse 20	2.14 % formaldehyde (solution 5)	0.22 ± 0
Corpse 21	1.83 % formaldehyde (solution 6)	0.18 ± 0.032
Corpse 22	1.48 % formaldehyde (solution 4)	0.13 ± 0.0058

Table 8: All corpses were positioned below the long throw nozzles, and were embalmed with reduced content of formaldehyde. Each corpse was treated with InfuTrace™ only on its surface, without reperfusion or injection into cavities. The samples were taken during skin removal. The formaldehyde exposure remained on a low level. (n=11)

**Table 9**: **Emissions of corpses embalmed with a common 3% formaldehyde perfusion and InfuTrace™** treatment with 100 mL injections each into thorax, abdomen, and multi-subcutaneously		
Corpse	*Dissection step*	*Formaldehyde exposure (ppm) ± SD*
Corpse 23	Skin opening	0.058 ± 0.0078
Corpse 24	Skin opening	0.036 ± 0.00058

Table 9: Two corpses were embalmed with common 3 % content of formaldehyde. Each corpse was treated with InfuTrace™ with 100 mL injections each into thorax, abdomen, and in addition multi-subcutaneously. The samples were taken during skin removal and using the long throw nozzles. The formaldehyde exposure fell far below 0.1 ppm. (n=12)

**Table 10: Formaldehyde exposure after successful reduction of Formaldehyde** emissions tested in a running dissection session with students, optimized InfuTrace™ treatment combined with long throw nozzles, corpses several months in use **(Fig. 9)**		
Corpse / dissection table	*Dissection step*	*Formaldehyde exposure (ppm)*
Corpse 16<br>Table 26, cachectic dry corpse	Muscle dissection	0.023 ± 0.0072
	During thorax opening	0.022 ± 0.0036
	Muscle dissection	0.023 ± 0.0015
	During abdomen opening	0.021 ± 0.00058
Corpse 17,<br>Table 23, very obese corpse, very moisty	Muscle dissection	0.026 ± 0.0065
	During thorax+abdomen opening	0.032 ± 0.0015
	Muscle dissection	0.028 ± 0.0057
Corpse 25<br>Table 24, corpse of common size	Muscle dissection	0.034 ± 0.011
	During thorax opening,	0.036 ± 0.010
Corpse 26<br>Table 18, corpse of common size	Muscle dissection	0.031 ± 0.0040
	During abdomen opening	0.034 ± 0.0045
	Muscle dissection	0.031 ± 0.0040
Corpse 27<br>Table 17, corpse of common size	Muscle dissection	0.034 ± 0.0052
	During thorax opening	0.030 ± 0.0032

Table 10: The final experiment with corpses embalmed with common 3 % content of formaldehyde: Each corpse was treated with InfuTrace™ (100 mL injections each into thorax, abdomen, and in addition multi-subcutaneously). The samples were taken below the long throw nozzles during muscle dissection, during thorax opening, during abdomen opening and - as a worst-case sceanario - during simultaneuosly opening thorax and abdomen in a running students course. The formaldehyde exposure remained far below 0.1 ppm even in critical dissection steps. (n=41)

**Table 11: Formaldehyde exposure measured by Radiello® passive-sampling versus active sampling**, in a running dissection lab **(Fig. 10)**			
*Person**	*Dissection table*	*Radiello® formaldehyde (ppm)*	*Active sampling formaldehyde (ppm)*
Person 1	Table 25 + 26	0.036	-
Person 2	Table 27 + 28	0.027	-
Person 3	Table 21 + 22	0.043	-
Person 4	Table 20 + 19	0.058	-
Person 5	Table 23 +24	0.045	0.032
Person 6	Table 17 + 18	0.042	0.025

*: teachers are responsible for two dissection tables and moved between these tables

Table 11: Formaldehyde exposure of teachers measured by passive and active sampling. All values were far below the German PEL and any “substance-index”. (n=8)

**Table 12:** Left: Formaldehyde exposure measured at common climate conditions (17°C/33% r.h.*)<br>Right: Formaldehyde exposure measured at a very hot summer day (20°C/78% r.h.*) seven weeks later		
Corpse / dissection table	*Formaldehyde (ppm) ± SD*<br>*17,7°C / 33% r.h.*	*Formaldehyde (ppm) ± SD*<br>*20°C / 78% r.h.*
Corpse 26, Table 18	0.023 ± 0.0028	0.056 ± 0.0078
Corpse 7, Table 26, cachectic little corpse	0.032 ± 0.0040	0.052 ± 0.0066

*: room temperature / relative humidity r.h.

Table 12: Formaldehyde exposure of teachers increased by about 40-60% with the high room temperature and extremely high relative humidity, but the values still remained far below the German PEL and any "substance-index". (n=27)

**Table 13: Sampling and chemical analysis by the Regierungspräsidum Kassel** of corpses which were in use for half a year and with optimized InfuTrace™-treatment and control corpse 30 without InfuTrace™-treatment and nozzles in work		
Corpse / dissection table	*Dissection procedure*	*Formaldehyde exposure (ppm) ± SD*
Corpse 28, Table 1	Skin dissection	0.038 ± 0,0068
Corpse 29, Table 2	Dissection of muscles	0.057 ± 0.019
Control corpse 30 ,Table 11	Skin dissection	0.24 ± 0.084

Table 13: Measurements by the governmental authority during skin dissection and dissection of muscles using long throw nozzles on corpses treated with InfuTrace™ that were half a year in use confirmed the low formaldehyde exposures with results below 0.1 ppm. The control corpse 30 without InfuTrace™- treatment resulted in much higher concentrations up to 0.32 ppm. (n=21)

Table 14: **Sampling and chemical analysis by the Regierungspräsidium Kassel** of corpses which were several months in use and with optimized InfuTrace™-treatment and nozzles in work		
Corpse / dissection table	*Dissection procedure*	*Formaldehyde exposure (ppm) ± SD*
Corpse 24 + 31, Table 1 + 2	Opening and dissection of the abdomen	0.019 ± 0.010
Corpse 23 + 32, Table 7 + 8	Opening and dissection of the abdomen	0.036 ± 0,0084
Corpse 11+ 33, Table 9 + 10	Opening and dissection of the abdomen	0.019 ± 0.0063
Corpse 29 + 34, Table 11 + 12	Opening and dissection of the abdomen	0.021 ± 0.0077

Table 14: Measurements by the governmental authority during opening and dissection of the abdomen using long throw nozzles on corpses treated with InfuTrace™ and several months in use confirmed the low formaldehyde exposures with results far below 0.1 ppm. (n=22)

**Table 15: Sampling and chemical analysis by Regierungspräsidium Kassel**, corpses have been a few weeks in use, and with optimized InfuTrace™-treatment and working nozzles		
Corpse / dissection table	*Dissection procedure*	*Formaldehyde exposure (ppm) ± SD*
Corpse 35 + 36, Table 3 + 4	Skin dissection and 7 dissection of fat tissue	0.056 ± 0.00071
Corpse 36, Table 4	Skin dissection and dissection of fat tissue	0.053 ± 0,0064
Corpse 37 + 38, Table 9 + 10	Skin dissection and dissection of fat tissue	0.046 ± 0.00071
Corpse 39, Table 11	Skin dissection and dissection of fat tissue	0.076 ± 0.0028
Corpse 40, Table 12	Skin dissection and dissection of fat tissue	0.089 ± 0.011

Table 15: Measurements by the governmental authority in a students´ course (skin dissection and dissection of fat tissue) using long throw nozzles on corpses treated with InfuTrace™ and only a few weeks in use confirmed the low formaldehyd exposures with results below 0.1 ppm. (n=12)

**Table 16: Graphical Summary** **Formaldehyde emissions before formaldehyde reduction** in a students´ dissection course without long throw nozzles and without InfuTrace™ treatment **(Fig. 11)**		
Corpse	*Dissection step*	*Formaldehyde exposure (ppm) ± SD*
Corpse 1	epifascial nerve dissection	1.1 0.82
Corpse 2	Inner cavities	0.71 ± 0.33
Corpse 3	Inner cavities	0.53 ± 0.11
Corpse 4	Inner cavities	0.83 ± 0.20
Corpse 5	epifascial nerve dissection	1.5 ± 0.86
Corpse 6	Skin opening	0.28 ± 0.17
**Table 16: Formaldehyde emissions were measured with long throw nozzles in use, without InfuTrace™ treatment**		
Corpse	*Dissection step*	*Formaldehyde exposure (ppm) ± SD*
Corpse 7	Inner cavities	0.21 ± 0.0071
Corpse 8	Inner cavities	0.30 ± 0.014
Corpse 9	Muscles	0.12 ± 0.00058
Corpse 10	Muscles	0.20 ± 0.070
**Table 16: Formaldehyde emissions after successful reduction of formaldehyde** tested in a running dissection session with attending students and optimized InfuTrace™ treatment combined with long throw nozzles		
Corpse	*Dissection step*	*Formaldehyde exposure (ppm) ± SD*
Corpse 11	Inner cavities	0.023 ± 0.0028
Corpse 12	Inner cavities	0.029 ± 0.0053
Corpse 13	Inner cavities	0.035 ± 0.0096
Corpse 14	Inner cavities	0.032 ± 0.0040
Corpse 15	Inner cavities	0.031 ± 0.0041
Corpse 16	Skin dissection	0.038 ± 0.0065
Corpse 17	Muscles	0.057 ± 0.019

Table 16: Graphical summary: Corpses 1-6 without InfuTrace™ treatment and without long throw nozzles in use; corpses 7-10 without InfuTrace™ treatment and with long throw nozzles in use; corpses 11-17 with optimized InfuTrace™ treatment and with long throw nozzles in use. Starting with formaldehyde exposures up to 1.5 ± 0.86 ppm the exposure finally did not exceed 0.057 ± 0.019 ppm. (Corpses 1-6 n=32; corpses 7-10: n=10, corpses 11-17: n=59)
